# The scholar role in the National Competence Based Catalogues of Learning Objectives for Undergraduate Medical Education (NKLM) compared to other international frameworks

**DOI:** 10.3205/000224

**Published:** 2015-11-12

**Authors:** Stefanie C. Hautz, Wolf E. Hautz, Niklas Keller, Markus A. Feufel, Claudia Spies

**Affiliations:** 1 Institut für Medizinische Lehre, Abteilung für Assessment und Evaluation, Medizinische Fakultät, Universität Bern, Switzerland; 2 Klinik für Anästhesiologie mit Schwerpunkt operative Intensivmedizin, Charité, Campus Mitte und Campus Virchow Klinikum, Berlin, Germany; 3 Universitäres Notfallzentrum, Inselspital Bern, Switzerland; 4 Prodekanat für Studium und Lehre, Charité Universitätsmedizin Berlin, Germany; 5 Max Planck Institut für Bildungsforschung, Harding Zentrum für Risikokompetenz, Berlin, Germany

**Keywords:** medical education, undergraduate education, scholarship

## Abstract

**Background:** In Germany, a national competence based catalogue of learning objectives in medicine (NKLM) was developed by the Society for Medical Education and the Council of Medical Faculties. As many of its international counterparts the NKLM describes the qualifications of medical school graduates. The definition of such outcome frameworks indents to make medical education transparent to students, teachers and society. The NKLM aims to amend existing lists of medical topics for assessment with learnable competencies. All outcome frameworks are structured into chapters, domains or physician roles. The definition of the scholar-role poses a number of questions such as: What distinguishes necessary qualifications of a scientifically qualified physician from those of a medical scientist?

**Methods:** 13 outcome frameworks were identified through a systematic three-step literature review and their content compared to the scholar role in the NKLM by means of a qualitative text analysis.

The three steps consist of (1) search for outcome frameworks, (2) in- and exclusion, and (3) data extraction, categorization, and validation. The results were afterwards matched with the scholar role of the NKLM.

**Results:** Extracted contents of all frameworks may be summarized into the components *Common Basics, Clinical Application, Research, Teaching and Education*, and *Lifelong Learning*. Compared to the included frameworks the NKLM emphasises competencies necessary for research and teaching while clinical application is less prominently mentioned.

**Conclusion:** The scholar role of the NKLM differs from other international outcome frameworks. Discussing these results shall increase propagation and understanding of the NKLM and thus contribute to the qualification of future medical graduates in Germany.

## Zusammenfassung

**Hintergrund:** In Deutschland haben die Gesellschaft für Medizinische Ausbildung (GMA) und der Medizinische Fakultätentag (MFT) den Nationalen Kompetenzbasierten Lernzielkatalog Medizin (NKLM) federführend entwickelt. Dieser beschreibt, wie viele internationale Pendants, detailliert Qualifikationen für Absolventen des Medizinstudiums. Die Definition derartiger Rahmenwerke folgt der Bestrebung, Ausbildungsinhalte den Lehrenden und Lernenden sowie der Gesellschaft transparent darzustellen.

Der NKLM ergänzt die Listen von Themen aus Gegenstandskatalogen durch eine Sammlung erlernbarer Kompetenzen. Alle Rahmenwerke sind dabei entweder in Kapitel oder Domänen oder nach ärztlichen Rollen gegliedert. Dabei wirft die Definition der Gelehrten-Rolle zahlreiche Fragen auf, wie Studien gezeigt haben: Welcher Unterschied besteht zwischen den notwendigen Qualifikationen als wissenschaftlich qualifizierter Arzt und denen als ärztlicher Wissenschaftler? Wie werden die Schwerpunkte gesetzt und wie unterscheiden sie sich im internationalen Vergleich?

**Methode:** In einer systematischen, dreistufigen Recherche wurden 13 internationale Rahmenwerke identifiziert und deren Inhalte durch eine qualitative Textanalyse mit den Inhalten der Gelehrten-Rolle des NKLM verglichen. Die drei Stufen umfassen (1) die systematische Suche, (2) die transparente Auswahl publizierter Rahmenwerke (in- und exclusion) und (3) die Datenextraktion, Kategorisierung und Validierung. Die Ergebnisse daraus wurden mit der Gelehrten-Rolle des NKLM verglichen.

**Ergebnisse:** Die extrahierten Inhalte aller Rahmenwerke lassen sich in die Komponenten *Gemeinsame Grundlagen, Klinische Anwendung, Forschung, Lehren und Bilden, sowie Lebenslanges Lernen* gliedern. Im Unterschied zu den verglichenen Rahmenwerken betont der NKLM die zum Forschen und Lehren notwendigen Kompetenzen, während die klinische Anwendung eine vergleichsweise untergeordnete Rolle spielt.

**Schlussfolgerung:** Die Inhalte der Gelehrtenrolle des NKLM unterscheiden sich von den Inhalten anderer internationaler Rahmenwerke. Die Diskussion der Ergebnisse soll zu Verbreitung und Verständnis des NKLM und damit zur Qualifikation zukünftiger Absolventen medizinischer Studiengänge in Deutschland beitragen.

## Introduction

In Germany, the contents of medical curricula are currently defined by the “Approbationsordnung für Ärzte” (ÄApprO) [1] and the EU-Directive 2005/36/EC [2] while those of the state exams are defined in the “Gegenstandskatalog” of the Institute of medical and pharmaceutical Exam Questions (IMPP) [3]. Because of the rather abstract directives of the ÄApprO, medical faculties and their teachers have a lot of room to manoeuvre.

Since 2009, the Society of Medical Education in Germany (GMA) and the Council of Medical Faculties (MFT) lead the process of developing a national, competence based catalogue of learning objectives for undergraduate medical education (NKLM) as commissioned by the conference of federal ministries of education [4]. The catalogue was consented in an extensive Delphi process including all 160 medical societies that are organized within the Association of the Scientific Medical Societies (AWMF) [5]. The NKLM lists the qualifications expected of graduates at the end of medical studies in detail and explicitly references comparable frameworks of other countries, especially the Canadian framework [6]. Since the development of the Canadian Medical Roles [6] in the 1990s, numerous countries have developed their own frameworks, some of which are based on the CanMEDS. Often, these frameworks reference each other [7]. Yet, the processes of development differ between these frameworks in important aspects: while the CanMEDS are based on a broad consensus process within the medical profession as well as within the general population [8], one framework established in Australia resulted from an initiative by deans and vice-deans of education of the medical faculties [9]. In Europe, next to national frameworks like the Swiss Catalogue of Learning Objectives [10], the Dutch Blueprint [11], the Scottish Doctor [12] and the British Tomorrow’s Doctors [13], the TUNING Project aims to harmonize aims, objectives, and contents of medical education within Europe [14]. 

All these frameworks are structured into chapters, domains or physician roles. The roles of the NKLM are structured along those of the CanMEDS [6] and contain: *Medical Expert, Communicator, Collaborator, Health Advocate, Manager, Professional*, and *Scholar*. Beyond this, the NKLM [4] contains the sections II (medical knowledge, clinical skills and professional attitudes) and III (patient centred prevention).

The *Scholar* role, however, raises a number of questions that have been identified in previous studies [15]: how many and which scientific qualifications does a graduate of medical studies need to be suitable for postgraduate training? What differences exist between the qualifications necessary for a scientifically working physician and a medical scientist? How do scientist and *Scholar* differ? 

In its chapter on the *Scholar*, the NKLM [4] provides answers to these questions with regards to Germany. But while every medical student exercises his roles of communicator, health advocate, or manager within the context and cultural setting of his own country, qualifications pertaining to the *Scholar* role are mostly not tied to national boundaries: “medical knowledge and research have traditionally crossed boundaries” [16]. 

The aim of the present study is therefore to compare the contents of the *Scholar* role of the NKLM to other national frameworks and identify commonalties and differences. Through discussion of the results among German physicians, and especially among persons interested in or responsible for medical education, we aim to contribute to the understanding and dissemination of the NKLM as well as to a critical and constructive dialogue with it and thus ultimately to the qualification of future graduates of medical studies in Germany.

## Method

The contents of the NKLM were compared to other published national frameworks in a three-step approach. The three steps consisted of 1) the systematic search, 2) a transparent selection of published frameworks based on pre-defined in- and exclusion criteria and 3) data extraction, categorisation and validation.

The systematic search was conducted by an experienced research librarian in June 2013 in MEDLINE, Embase and Google using the search terms: medical education, outcome framework, learning objective, government*, curriculum and combinations thereof. The detailed strategy is available upon request. All results from MEDLINE and Embase as well as the first one hundred Google results were saved in EndNote. Further frameworks were extracted from references of the results as well as from the authors’ collections. 

**Figure 1:**
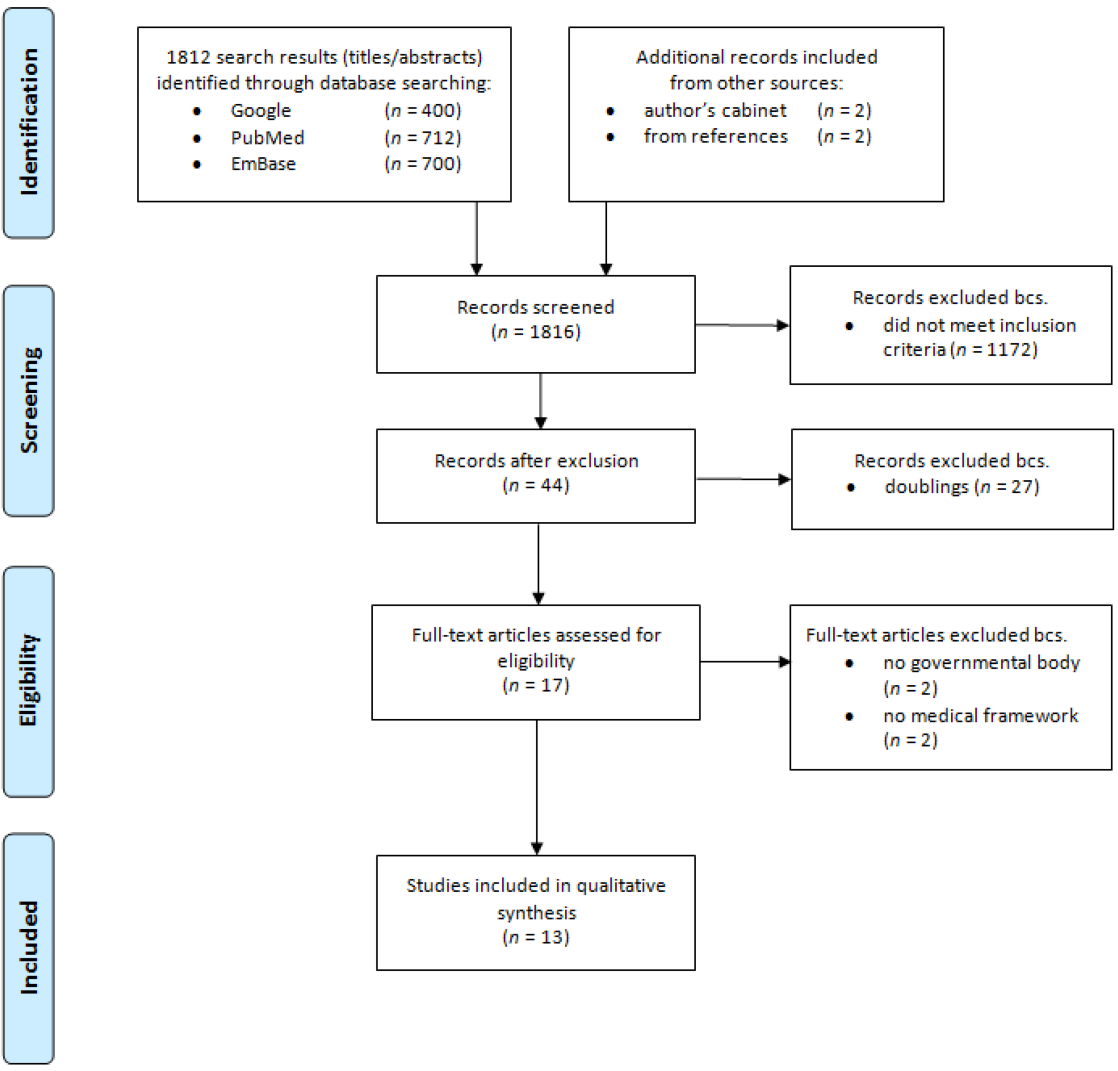
Search, selection and inclusion of frameworks used according to the PRISMA-Standard [25]

All frameworks resulting from a national consensus process and endorsed or published by a national medical society or governmental office were included for a full text analysis after exclusion of duplicates. Of those, accreditation standards, frameworks of medical sub-disciplines, inter-professional frameworks, and frameworks developed by students were excluded. All in- and exclusions were conducted by two authors based on these criteria independently. Conflicts were resolved by discussion and consensus. The complete search and selection strategy is depicted in Figure 1 (Fig. 1).

Data were extracted from the 13 included frameworks in two steps. First, the contents of the *Scholar*-section of all seven frameworks containing such a section were included in full and added to a generic collection. This collection was compared to the full text of the remaining six frameworks and all content considered similar or equivalent was added to the collection. As above, this process was conducted by two authors independently and all conflicts were resolved by discussion and consensus. The source of every piece of content added to the collection – typically a sentence or half-sentence – remained traceable at all times (see [17]). 

To compare the *Scholar* role of the NKLM to the contents of this collection, all contents of the collection were compared to those of the NKLM by two independent raters. All objectives from the collection and the NKLM were grouped into inductively generated categories by means of an inductive discourse (for methodological details see [7]). Next, we generated a comparison of each objective of the collection to those of the *Scholar* role within the NKLM. In order to assess the quality of the assignments to the categories we calculated an intra-class correlation of the raters’ assignments (see results). 

## Results

The search yielded a total of 1,816 articles, of which 13 were included as outcome frameworks (see Figure 1 (Fig. 1)). Of those, seven contain a role, chapter or domain entitled *Scholar* (see Table 1 (Tab. 1)). The definitions of the *Scholar* role differ significantly in terms of extent, content and level of detail.

The *Scholar* role in international frameworks may be split into five components that in total contain all of the contents of the role while at the same time each containing contents from all analysed frameworks. These components encompass *common basics*, *clinical application*, *research*, *teaching* *and educating*, as well as *lifelong learning* [17]. The NKLM itself structures the *Scholar* (containing a total of 47 learning objectives) into the “lifelong learner” (8 objectives), the “critical applier” (11), the “teacher/tutor” (13) and the “innovator” (11). The contents under these headings only slightly correspond to the components resulting from the comparison of the international frameworks. 

Extending one’s view beyond the *Scholar*-chapter to include all roughly 2,500 learning objectives of the NKLM, one can identify 95 objectives (from the *Scholar* role and the chapter on medical-scientific skills), that can be assigned to one of the five components of the *Scholar:* *common basics*, *clinical application*, *research*, *teaching and educating* or *lifelong learning*. Only two objectives cannot be assigned. The remaining 93 objectives concern research in 57% (see Table 2 (Tab. 2)). Inter-rater agreement in selecting and assigning learning objectives was good to excellent (ICC(2,1) = 0.907). 

The number of learning objectives in Section I (roles) of the NKLM within the *Scholar* role is 47 and thus presents an average with regards to all roles and objectives (17%, 7 roles). In section II, the number of objectives in the chapter medical-scientific skills presents 3% of all objectives listed within (48 objectives of a total of 1,649).

### Common Basics

The component common basics resulting from international frameworks contains: scientific attitude, critical thinking, and basic knowledge of focused information search. These components are required in order to competently exercise the four other components. Exemplary quotes from international frameworks like “Critically evaluate medical information and its sources, and apply this appropriately to practice decisions” [6] or “Demonstrates a commitment to excellence, evidence based practice and the generation of new scientific knowledge” [18] are equated to objectives in the NKLM such as: “[Graduates] search, find and interpret relevant sources in an appropriate way. They are able to research relevant (secondary and tertiary) literature and other information sources with appropriate researching systems and effective search strategies and make a selection, read and interpret” [4]. 

### Clinical Application

The ability to clinically apply scientific results and methods is extensively required by all included international frameworks in great detail. The NKLM list comparable objectives only in part.

The quote “The physician demonstrates up-to-date knowledge of the evidence-based standard of medical care for common ailments and illnesses and the basic urgent interventions.” [10] is partly equated in the NKLM as “[Graduates] are able to take evidence based decisions and critically evaluate data – also connecting them to the available evidence” [4]. Also the aim “The doctor is able to approach a health problem in a systematic way using theories and hypothesis formation and problem solving, models and decision theories” [10] is partly picked up by the NKLM as “[Graduates] integrate conclusions that are based on critical appreciation into their everyday work. They are able to methodically question their own doing” [4].

Yet no real equivalence can be found in the NKLM’s *Scholar* role for the objectives “Applies epidemiological data managing the health of patients and their communities” [10], “Applies the concept of specificity, sensitivity, pre and post-test probability to the interpretation of common diagnostic procedures” [9] and “You must be familiar with the guidelines and developments that effect your work” [19].

### Research

The competencies necessary to conduct research oneself, take up the largest space within the NKLM’s *Scholar* role in terms of quantity and are also qualitatively more differentiated compared to the other included frameworks (see Table 2 (Tab. 2)). While the description of the required competencies is limited in detail to “Knowledge and appreciation of quantitative and qualitative methodologies, including the differences between them, and their appropriate usage” [12] in the international frameworks, the NKLM requires graduates to “reflect upon the historical developments of experiment, animal and human experiment and the epistemology of medical research” and to be able “to identify the differences between descriptive, empirical-experimental, mathematical and hermeneutic-interpretive methods of knowledge creation” [4]. 

Many frameworks – including the NKLM – require knowledge of study design, ethical boundaries of research and statistical methods. Next to the NKLM however only two frameworks [6], [11] require graduates to be able to “disseminate their own research results in an appropriate way”. 

### Teaching and Educating

Competences necessary for teaching are described in much greater extend both quantitatively as well as qualitatively in the NKLM than in comparable frameworks. They require from graduates to “recognize the value of teaching and training others” [20]. The NKLM, in comparison, writes: “[Graduates] know how to apply importand methods of teaching and learning in adult education as well as methods for assessment, judgement and evaluation. […] They are able to convey learning contents to patients or relatives by means of a consultation, lecture or in other appropriate ways” [4].

### Lifelong Learning

The ability for lifelong learning is described in the NKLM on a level much less abstract then in the other included frameworks. While internationally these frameworks state that “As learners, they recognize the need to be continually learning and model this for others” [6] or “You must keep your professional knowledge and skills up to date” [19], the NKLM clearly states what it means by lifelong learning: “[Graduates] master the principles of learning in a sense that enables recognition of, reflection on, and attainment of their own learning need as well as the application of learning results”. “They are able to take responsibility for continuous education for ongoing retention and development of physician competencies by adequately recognizing their state of development in all areas of competence, judging it, and taking appropriate measures where necessary” [4].

## Discussion

Through a qualitative text analysis the *Scholar* role of the NKLM was compared to 13 international outcome frameworks. In doing so we partially identified commonalities as well as important differences with regard to the components of the scholar role identified in international frameworks [17]. One distinguishing feature of the *Scholar* role in the NKLM as well as in the international frameworks is the fact that it bundles together many different competencies such as evidence based clinical decision making or competent teaching and assessment, while other roles such as *Communicator* are limited to communication competencies only. Along with the few examples of the application of the role, this may explain why the *Scholar* role is judged as difficult by educators and not well understood [15]. 

Comparing the contents of the *Scholar* role within the NKLM to international frameworks identifies an emphasis on research in the NKLM, while other frameworks focus mainly on clinical application. While the objectives of the NKLM in the area of common basics are comparable in terms of number and extend to those of many other frameworks, the requirements in teaching and education are much more detailed and extensive than in the frameworks analysed herein. 

A fervent discussion exists on the necessity, depth and extent of scientific qualifications of a medical graduate [5], [21]. While already Humboldt in his ideals [22], as well as other authors [23] implicated a connection between the quality of research and education, there is almost no empirical evidence on this issue [24]. Newer data in educational research further suggest that extensive knowledge of biomedical basics in medicine limit the acquisition of clinical knowledge and skills rather than supporting it [23], something the authors attribute to the differences in cognitive processes for the acquisition and application of both types of knowledge.

## Conclusion

The *Scholar* role in the NKLM as compared to numerous other national outcome frameworks emphasises competencies required for research and teaching while critical clinical application of scientific results is of comparatively lower importance. Thus, the NKLM differs in important ways from other international frameworks and clearly emphasises a methods-oriented education of physicians as researchers.

## Notes

### Competing interests

The authors declare that they have no competing interests.

### Authorship

The authors Hautz SC and Hautz WE contributed equally to this article.

## Figures and Tables

**Table 1:**
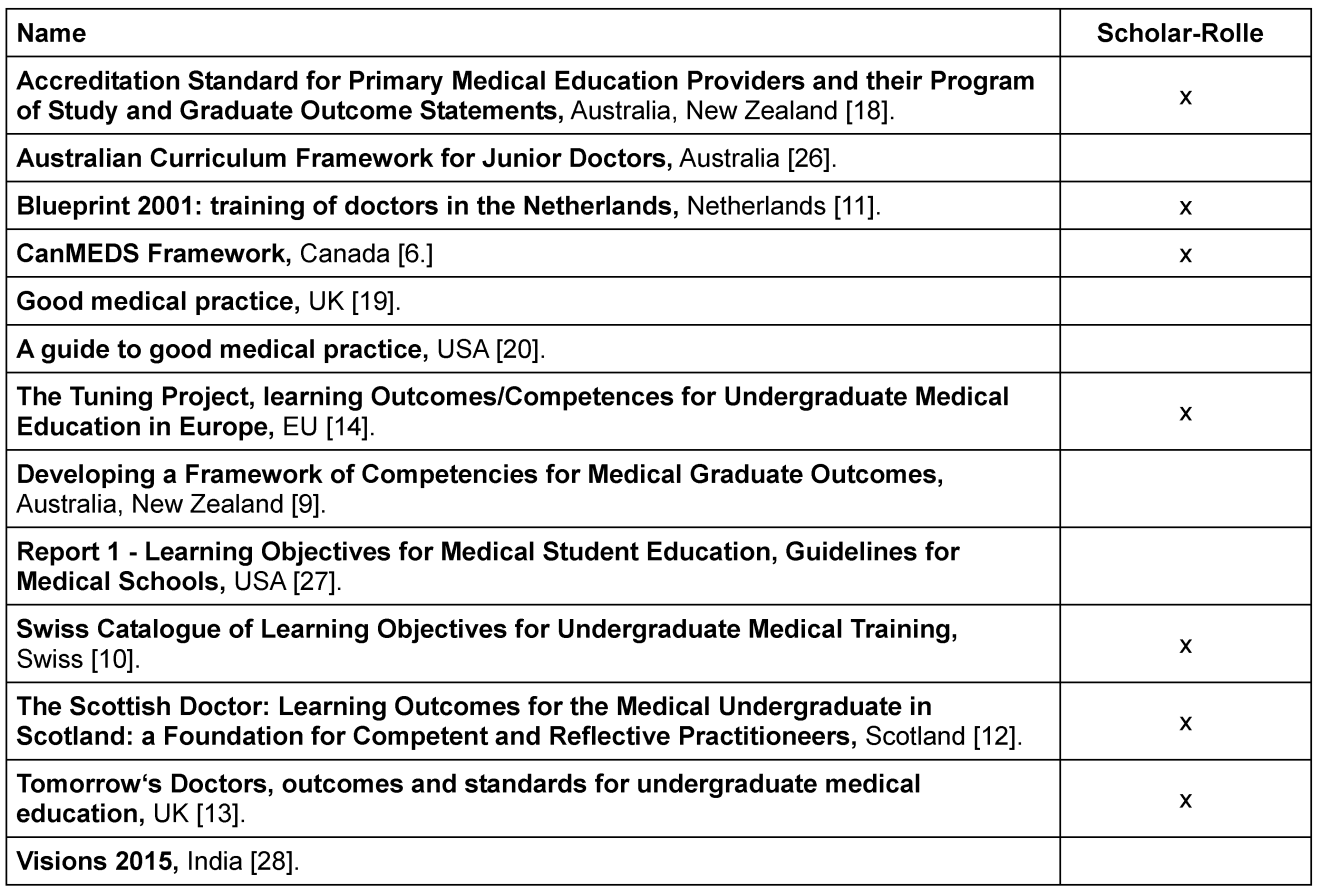
Frameworks included

**Table 2:**
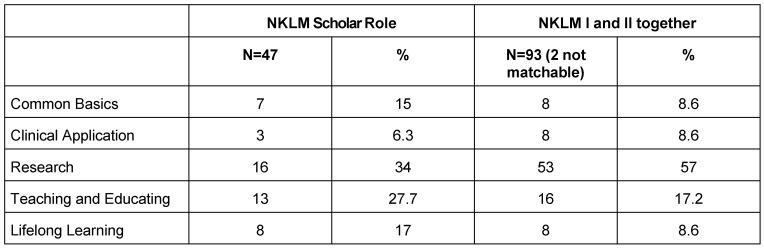
Assignment of learning objectives from the chapter Scholar as well as within the full NKLM to one of the five components as extracted from the other frameworks. Some objectives are assigned to more than one component. The absolute number of assigned objectives is given in each column-heading.

## References

[1] (2013). Approbationsordnung für Ärzte ÄApprO 2002. Approbationsordnung für Ärzte vom 27. Juni 2002 (BGBl. I S. 2405), die zuletzt durch Artikel 2 der Verordnung vom 2. August 2013 (BGBl. I S. 3005) geändert worden ist.

[2] (2005). Directive 2005/36/EC of the European Parliament and of the Council of 7 September 2005 on the recognition of professional qualifications. Official Journal of the European Union.

[3] (2005). IMPP Gegenstandskataloge.

[4] MFT Medizinischer Fakultätentag der Bundesrepublik Deutschland e.V. (2015). Nationaler Kompetenzbasierter Lernzielkatalog Medizin (NKLM).

[5] Fischer MR, Bauer D, Mohn K, NKLM-Projektgruppe (2015). Finally finished! National Competence Based Catalogues of Learning Objectives for Undergraduate Medical Education (NKLM) and Dental Education (NKLZ) ready for trial. GMS Z Med Ausbild.

[6] Frank JR (2005). The CanMEDS 2005 physician competency framework. Better standards. Better physicians. Better care.

[7] Hautz SC, Hautz WE, Feufel MA, Spies CD (2015). Comparability of outcome frameworks in medical education: Implications for framework development. Med Teach.

[8] (2000). CanMEDS 2000: Extract from the CanMEDS 2000 Project Societal Needs Working Group Report. Med Teach.

[9] Carmichael A, Hourn M (2011). Developing a Framework of Competencies for Medical Graduate Outcomes.

[10] Working Group under a Mandate of the Joint Commission of the Swiss Medical Schools (2008). Swiss Catalogue of Learning Objectives for Undergraduate Medical Training.

[11] Metz JCM, Verbeek-Weel AMM, Huisjes HJ (2001). Blueprint 2001: training of doctors in The Netherlands. Ajusted objectives of undergraduate medical education in The Netherlands.

[12] Scottish Deans' Medical Education Group (2008). The Scottish Doctor: Learning Outcomes for the Medical Undergraduate in Scotland: a Foundation for Competent and Reflective Practitioneers.

[13] (2009). Tomorrow's Doctors. Outcomes and standards for undergraduate medical education..

[14] Cumming A, Ross M (2008). The Tuning Project (medicine) – learning outcomes/competences for undergraduate medical education in Europe.

[15] Wiechers S, Durante S, Lammerding-Köppel M (2013). Verständnis der Kompetenzrollen des Nationalen Kompetenzbasierten Lernzielkataloges Medizin (NKLM) bei Lehrenden. http://dx.doi.org/10.3205/13gma174.

[16] Core Committee, Institute for International Medical Education (2002). Global minimum essential requirements in medical education. Med Teach.

[17] Hautz SC, Hautz WE, Feufel MA, Spies CD What makes a doctor a scholar. A systematic review and content analysis of outcome frameworks. BMC Med Educ.

[18] Australian Medical Council Limited (2012). Standards for Assessment and Accreditation of Primary Medical Programs by the Australian Medical Council 2012.

[19] General Medical Council (2013). Good medical practice.

[20] (2009). A Guide to Good Medical Practice - USA. Version 1.1.

[21] Ziemann E, Oestmann JW (2012). Publications by doctoral candidates at Charité University Hospital, Berlin, from 1998-2008. Dtsch Arztebl Int.

[22] Ifenthaler D, Gosper M, Gosper M, Ifenthaler D (2014). Research-based learning: Connecting research and instruction. Curriculum models for the 21st Century. Using learning technologies in higher education.

[23] Schauber SK, Hecht M, Nouns ZM, Dettmer S (2013). On the role of biomedical knowledge in the acquisition of clinical knowledge. Med Educ.

[24] Hattie J, Marsh HW (1996). The relationship between research and teaching: a meta-analysis. Rev Educ Res.

[25] Moher D, Liberati A, Tetzlaff J, Altman DG, PRISMA Group (2009). Preferred reporting items for systematic reviews and meta-analyses: the PRISMA statement. PLoS Med.

[26] ACF Revision Working Group (2009). Australian Curriculum Framework for Junior Doctors. Version 2.2.

[27] (1998). Report I - Learning Objectives for Medical Student Education Guidelines for Medical Schools. Medical School Objectives Project.

[28] Medical Council of India (2011). Visions 2015.

